# Redox-sensitive carrier-free nanoparticles self-assembled by disulfide-linked paclitaxel-tetramethylpyrazine conjugate for combination cancer chemotherapy

**DOI:** 10.7150/thno.42260

**Published:** 2021-02-20

**Authors:** Liang Zou, Xiaowei Liu, Jingjing Li, Wei Li, Lele Zhang, Chaomei Fu, Jinming Zhang, Zhongwei Gu

**Affiliations:** 1 Key Laboratory of Coarse Cereal Processing of Ministry of Agriculture and Rural Affairs, Chengdu University, Chengdu 610106, People's Republic of China.; 2 State Key Laboratory of Southwestern Chinese Medicine Resources, College of Pharmacy, Chengdu University of Traditional Chinese Medicine, Chengdu 611137, People's Republic of China.; 3 School of Basic Medical Sciences, Chengdu University, Chengdu 610106, People's Republic of China.; 4 Department of Pharmacology and Pharmacy, University of Hong Kong, Hong Kong 999077, People's Republic of China.; 5 College of Materials Science and Engineering, Nanjing Tech Universit`y, Nanjing 211816, People's Republic of China.

**Keywords:** drug conjugate, paclitaxel, tetramethylpyrazine, combination chemotherapy, disulfide linkage, self-assembly.

## Abstract

**Rationale:** Combinations of two or more therapeutic agents targeting different signaling pathways involved in tumor progression can have synergistic anticancer effects. However, combination chemotherapies are greatly limited by the different pharmacokinetics, tumor targeting, and cellular uptake capacities of the combined drugs. We have previously demonstrated the potential synergistic efficacy of paclitaxel (PTX) and the natural anti-angiogenic agent tetramethylpyrazine (TMP) for suppressing ovarian carcinoma growth. An efficient, facile, and smart nanosystem to deliver PTX and TMP simultaneously *in vivo* is greatly desired.

**Methods:** We constructed a redox-sensitive nanosystem based on the amphiphilic PTX-ss-TMP conjugate, in which PTX and TMP are linked by a disulfide bond. We characterized the structure of the drug conjugate by ^1^H NMR and LC-MS, and then prepared PTX-ss-TMP NPs by a one-step nanoprecipitation method. We investigated the redox sensitivity, tumor-targeting ability, anticancer efficacy, and anti-angiogenesis activity of PTX-ss-TMP NPs *in vitro* and *in vivo*.

**Results:** The amphiphilic PTX-ss-TMP conjugate readily self-assembled into stable nanoparticles in aqueous solution with a low critical association concentration of 1.35 µg/mL, well-defined spherical structure, small particle size (152 nm), high drug loading, redox-responsive drug release, high biocompatibility, and high storage stability. In cancer cells pretreated with GSH-OEt, PTX-ss-TMP NPs exhibited higher cytotoxicity, apoptosis rate, and cell-cycle arrest than monotherapy or combination therapy with free drugs, which was attributed to their improved cellular uptake and rapid intracellular drug release. Additionally, PTX-ss-TMP NPs also had a stronger anti-angiogenesis effect in HUVECs than free drug, which was mediated by VEGFR2-involved downstream signals. Finally, PTX-ss-TMP NPs showed tumor-specific accumulation and excellent antitumor activity in A2780 xenograft mice compared with free drug.

**Conclusions:** These *in vitro* and *in vivo* results provide clear evidence that this redox-responsive carrier-free nanosystem with intrinsic amphiphilicity has great potential for combination cancer chemotherapy.

## Introduction

Conventional mono-chemotherapy is greatly impeded by the physiological complexity of tumors, which causes poor treatment efficacy, multidrug resistance, tumor recurrence, and metastasis 1, 2. Recently, combination chemotherapy has been used in the clinic to address these challenges 3. Simultaneous administration of two or more therapeutic agents modulates multiple signaling pathways involved in tumor progression 4, 5, which may induce synergetic responses, reduce drug resistance, and mitigate side effects. Typically, agents that damage or intercalate DNA, such as doxorubicin 6, cisplatin 7, and gemcitabine 8, are combined with paclitaxel (PTX). The antitumor effects of PTX have also been enhanced by combinations with agents such as the P-glycoprotein inhibitor tetrandrine 9, the microtubule-associated inhibitor combretastatin A4 10, and the mTOR inhibitor everolimus 11.

Recently, anti-angiogenic agents have also been shown to synergistically improve the therapeutic outcomes of cytotoxic drugs, since angiogenesis supports tumor growth 12, 13. For example, the first anti-VEGF agent Bevacizumab (humanized monoclonal antibody) and plasmid expressing interfering RNA targeting VEGF (shVEGF) have been used in combination therapy 14. Another potential agent for combination chemotherapy is Ligustrazine (2,3,5,6-tetramethylpyrazine, TMP), a purified component of Rhizoma Chuanxiong that has been widely used to treat cerebral and cardiac ischemic diseases 15. TMP has also shown some benefits for tumor treatment, including reversal of multidrug resistance 16, suppression of metastasis and angiogenesis 17, and induction of apoptosis 18. In our previous study, we combined PTX and TMP to overwhelmingly suppress tumor growth in ovarian carcinoma 19. However, the combination therapy regime was far from ideal due to the different pharmacokinetics of the drugs and their nonspecific biodistributions and membrane transport properties 20.

Co-loading multiple drugs in a single nanocarrier guarantees that they are delivered to the tumor simultaneously and can improve their accumulation in tumor tissue relative to non-cancerous sites. Currently, various nano-carriers such as liposomes, polymeric nanoparticles, micelles, and dendrimers have been utilized to co-deliver combination chemotherapies 21. However, these sophisticated nanocarriers still suffer from some limitations, for instance, unreliable/unstable encapsulation of multiple drugs 22, the low drug loading capacity (typically <10%) 23, complicated polymer fabrication 24, potential toxicity 25, and the materials degradation/metabolism concerns 26. To resolve these problems, several amphiphilic drug-drug conjugates have been developed, such as irinotecan-chlorambucil 27, doxorubicin-ss-doxorubicin 23, and irinotecan-ss-quinine 28.

As proof of concept, we constructed a redox-sensitive nanosystem based on the amphiphilic PTX-ss-TMP conjugate, in which PTX and TMP are linked through a disulfide bond. This nanosystem design has several potential advantages. Primarily, both PTX and TMP can be fully encapsulated into nanomicelles via self-assembly of the PTX-ss-TMP conjugate and simultaneously transported to the tumor site. This carrier-free co-delivery system also avoids use of potentially toxic or inert carrier materials. The PTX-ss-TMP nanoparticles (NPs) should be stable in circulation and then rapidly disintegrate in tumor tissue in response to the much higher intracellular concentration of glutathione (GSH) than that in normal tissue. This dramatic drug release by redox-sensitive GSH stimulation would selectively kill tumor cells. Moreover, tumor progression may be effectively suppressed by the combined effects of PTX and TMP on cancer cell proliferation arrest and angiogenesis inhibition, respectively.

## Materials and Methods

### Materials

PTX (purity > 95%), D-α-tocopherol polyethylene glycol succinate (TPGS), and GSH were purchased from Dalian Meilun Biotechnology Co., LTD (Dalian, China). TMP (purity > 98%), thiodiglycolic anhydride, 4-dimethylaminopyridine (DMAP), *N*,*N*′-dicyclohexylcarbodiimide (DCC), and *N*-hydroxysuccinimide (HOSu) were obtained from Saeng Chemical Co., Ltd (Shanghai, China). Acetic anhydride, acetic acid, and hydrogen peroxide were obtained from Kelong Chemical Co., Ltd (Chengdu, China). DMSO-*d_6_* and trichloromethane-*d_6_* were purchased from Sigma-Aldrich (Shanghai) Trading Co., Ltd. Glutathione monoethyl ester (GSH-OEt) was obtained from Adipogen, Life Sciences, USA. Other chemicals and reagents were of analytical grade and obtained commercially.

The human ovarian cancer cell lines A2780 and SKOV3 were supplied by American Type Culture Collection (ATCC, USA). Cells were cultured using DMEM medium supplemented with 10% fetal bovine serum (FBS), 100 U/mL penicillin, and 100 μg/mL streptomycin in a humidified incubator containing 5% CO_2_ at 37 ºC. DMEM culture medium, FBS, PBS, penicillin, and streptomycin were purchased from Gibco, ThermoFisher Scientific, USA. Human umbilical vein endothelial cells (HUVECs) were obtained from Invitrogen (Carlsbad, CA, USA) and cultured in Kaighn's modification of Han's F12 medium (F-12K) complete media with 100 µg/mL heparin, 30 µg/mL ECGS, 10% heat-inactivated FBS and 1% penicillin-streptomycin.

Female nu/nu nude mice were supplied by the Experimental Animal Center of Chengdu Dashuo Biotechnology Co., Ltd. (Chengdu, China). Mice were maintained with free access to food and water and housed in a temperature-controlled barrier facility on a 12 h light and 12 h dark cycle. All animal experiments were approved by the Institutional Animal Care and Ethics Committee of Chengdu University.

### Synthesis of PTX-ss-TMP conjugate

Reactions were monitored by thin layer chromatography using silica gel-coated aluminum sheets (Qingdao Haiyang Chemical Co., Qingdao, China) and visualized by UV light (254 nm). The precipitates were purified by silica gel column chromatography using 200-300 mesh silica gel. The yields were calculated based on the last reaction step. The structures of all generated derivatives were confirmed by ^1^H NMR and ^13^C NMR assays using a JNM-ECZ600R/S_1_ NMR spectrometer (JEOL, Japan), UV-vis absorption spectroscopy, and high-resolution mass spectrometry analysis on an ultrafleXtreme MALDI-TOF (Bruker, Germany).

### Synthesis of 2-(bromide-methyl)-3,5,6-trimethylpyrazine

**Figure 1:**
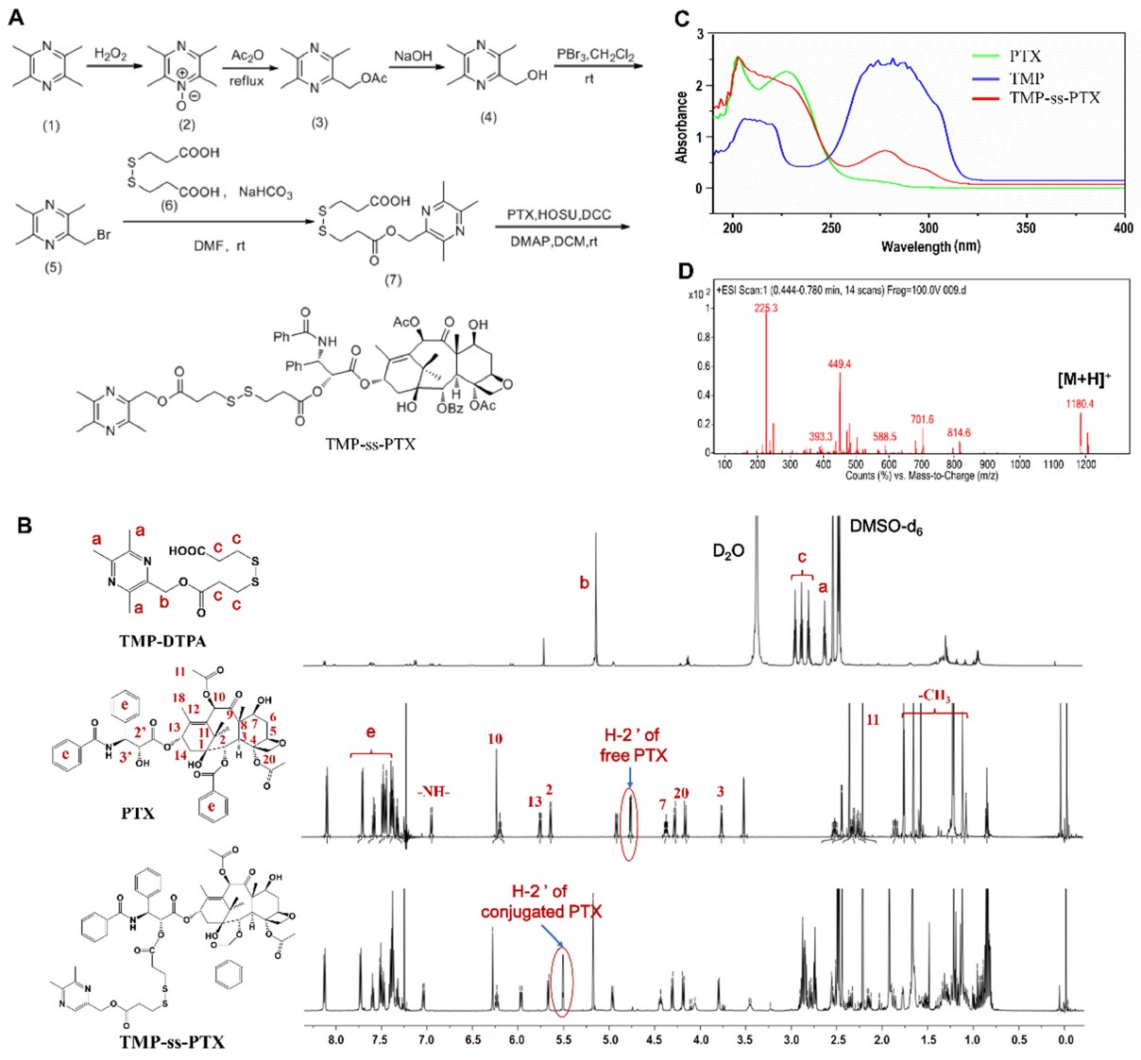
** Synthesis and characterization of PTX-ss-TMP conjugates. (A)** Synthetic routes of PTX-ss-TMP. **(B)** ^1^H NMR spectra (DMSO-*d_6_* as solvent) of PTX-ss-TMP and its synthetic intermediates. **(C)** UV-vis absorption spectra of PTX, TMP, and PTX-ss-TMP. **(D)** ESI/MS spectrum of PTX-ss-TMP.

First, TMP was dissolved in acetic acid and reacted with 30% H2O2 (1:1.1 molar ratio) at 70 °C for 4 h. The reactants were extracted by dichloromethane (DCM) to obtain TMP monoxides (shown in **Figure 1A** as chemical **2**). Subsequently, the TMP monoxides were dissolved in acetic anhydride and refluxed for 3 h at 120 °C. The reaction was terminated by addition of NaOH solution and then extracted with DCM. The DCM mixture was filtered and evaporated under vacuum. 2-Acetoxy-3,5,6-trimethylpyrazine (**3**) was purified and obtained by silica gel column chromatography as a pale green oil product. The intermediate (3,5,6-trimethylpyrazin-2-yl)methanol (**4**) was obtained by hydrolysis in NaOH solution with stirring for 1 h. Then, the intermediate (**4**) was further reacted with phosphorus tribromide (PBr_3_) in DCM for 1 h with stirring at 0 ºC to yield the intermediate 2-(bromide-methyl)-3,5,6-trimethylpyrazine (**5**). The reaction mixture was poured into ice-water and the crude product was extracted with ethyl acetate. After drying the organic layer over anhydrous Na2SO4 and evaporating the solvent under vacuum, the crude product was purified by silica gel column chromatography as white crystals (yield 83-91%).

### Synthesis of TMP-DTPA conjugate

A TMP derivative containing a disulfide linkage (**7**) was produced by an alkylation reaction. The intermediate 2-(bromide-methyl)-3,5,6-trimethylpyrazine (**5**) was reacted with 3,3'-dithiodipropionic acid (DTPA) and NaHCO_3_ (1:1:3 molar ratio) in *N*, *N*-dimethylformamide (DMF) with stirring for 3 h at 25 ºC. The crude TMP-DTPA conjugates were purified by silica gel column chromatography as yellowish oil products (yield 75-85%).

### Synthesis of PTX-ss-TMP conjugate

The disulfide-containing PTX-TMP conjugate (PTX-ss-TMP) was synthesized through a condensation reaction between PTX and TMP-DTPA using HOSu, DCC as a condensing agent, and DMAP as a catalytic agent. TMP-DTPA (1 mmol) was dissolved in dry DMF then added to HOSu (2 mmol) and DCC (1.5 mmol) using a syringe. After 4 h of activation, PTX (0.5 mmol) and DMAP (1 mmol) were added. The condensation reaction was maintained at 25 °C for 24 h. Then, the crude product was dialyzed against DMF to remove unreacted small molecules and separated by silica gel column chromatography to yield the purified PTX-ss-TMP conjugate (yield 77-89%).

### Preparation and characterization of PTX-ss-TMP NPs

PTX-ss-TMP NPs were prepared by a one-step nanoprecipitation method. 10 mg PTX-ss-TMP was dissolved in 1 mL DMSO. Then, the mixture was injected dropwise into 5 mL 0.03% TPGS with slight stirring (300 rpm) for 0.5 h. Next, the dispersion was transferred into dialysis tubes (MWCO 2000 Da) and dialyzed against 3 L of deionized water for 4 h. The dialysis medium was refreshed every 0.5 h to remove residual DMSO and emulsifier. Finally, the volume of the dispersion was increased to 10 mL by addition of PBS for a PTX-ss-TMP NPs concentration of 1 mg/mL for further experiments.

### Nanoparticle characterizations

Particle size, polydispersity index (PDI), and zeta potential were measured by dynamic light scattering (DLS) and electrophoretic light scattering using a Malvern Zetasizer Nano ZS (Malvern. U.K.). The particle morphology was observed by transmission electron microscopy (TEM, FEI Tecnai G2 20, USA) operated at an accelerating voltage of 200 kV with 2% phosphotungstic acid staining.

### Redox sensitivity test

Freshly prepared PTX-ss-TMP NPs were incubated with 0 or 10 mM GSH at 37 ºC for 24 h then assessed by DLS, TEM, and HPLC-TOF/MS. *In vitro* drug release from PTX-ss-TMP NPs in the presence of GSH (10 µM and 10 mM) was tested using a dialysis method under simulated physiological conditions (pH 7.4, 37 °C). 2 mL PTX-ss-TMP NPs was added to a dialysis bag (MWCO 1000 Da), which was immersed into 40 mL buffer medium with GSH and kept at 37 °C in a shaker (100 rpm). At predetermined intervals, 2 mL of the external buffer was withdrawn and replaced with fresh medium. The amounts of released PTX and TMP at each timepoint were measured by HPLC.

### Critical aggregation concentration (CAC) determination of PTX-ss-TMP

The CAC of PTX-ss-TMP was determined using pyrene as a fluorescent probe 29. A series of concentrations of PTX-ss-TMP (0.05-50 µg/mL) were prepared, containing 6 × 10^-6^ M pyrene. The fluorescence emission of the samples was measured with excitation at 335 nm using a fluorescence spectrophotometer (Shimadzu RF-5301, Japan). The CAC was estimated as the inflection point in the plot of PTX-ss-TMP concentration versus emission intensity ratio I_373_/I_384_.

### Hemolysis assay

Membrane disruption of red blood cells (RBCs) was employed to evaluate the hemolytic effect of PTX-ss-TMP NPs 30. RBCs from healthy rabbits were collected according to the published procedure. Then, 1 mL water or saline was added to 1 mL RBCs suspension as the positive and negative hemolysis controls, respectively. Correspondingly, 1 mL NPs was mixed with 1 mL RBCs suspension. The samples were incubated at 37 ºC for 4 h, then the supernatant was withdrawn to detect hemoglobin leakage by optical density (OD) at 540 nm. The hemolysis rate of the NPs was calculated by the following equation: Hemolysis rate (%) = (OD_NPs_ - OD_neg_) / (OD_pos_ - OD_neg_) × 100.

### Storage stability assessment

The *in vitro* storage stability of PTX-ss-TMP NPs in PBS (pH 7.4) and RPMI 1640 media containing 5%, 10%, or 20% FBS was evaluated by DLS after 1, 5, and 10 days of incubation.

### *In vitro* cytotoxicity assay

The cytotoxicity of PTX-ss-TMP NPs to A2780 and SKOV3 cells was evaluated by MTT assay. The cells were seeded on 96-well dishes at 5 × 10^3^ cells per well and allowed to attach overnight. To mimic the high redox environment of tumor tissue, the cells were also preincubated with GSH-OEt for 2 h 23. The cells were then treated with various concentrations of TMP, PTX, PTX+TMP mixture, or PTX-ss-TMP NPs in culture media for 48 h. Then, 5 mg/mL MTT was added and the generated formazan crystals were dissolved in 100 μL DMSO. The absorbance at 540 nm in each well was measured with a 96-well plate reader, and cell viability was expressed as a percentage of untreated control.

### Cell apoptosis, cell cycle arrest and western blotting analysis

A2780 cells were cultured in 12-well dishes (5 × 10^4^ cells/well) overnight then treated with 100 nM TMP, PTX, PTX+TMP mixture, or PTX-ss-TMP NPs for 48 h. A group of cells was also preincubated with GSH-OEt for 2 h prior to PTX-ss-TMP NPs addition. Next, the cells were collected by trypsinization, rinsed, resuspended in binding buffer, and double stained with an annexin V-fluorescein isothiocyanate (FITC)/propidium iodide (PI) kit. Cell apoptosis was measured by flow cytometry (FCM) using a BD FACSCalibur flow cytometer according to the manufacturer's instructions. A total of 1 × 10^4^ gated events were recorded per sample.

As PTX is known to destroy microtubules, cell cycle distribution was also evaluated by FCM. Upon completion of the above treatments, the cells were harvested by trypsinization, fixed in 70% ethanol, and incubated overnight at 4 °C. Next, the cells were treated with RNase A, stained with PI (1 mg/mL), and analyzed by FCM.

Protein expression following the above treatments was also assessed by western blotting. Equivalent concentrations of protein were separated by electrophoresis on SDS-PAGE gels blocked with 5% skimmed milk. The membranes were incubated with primary antibodies related to cell apoptosis, including anti-PARP, cleaved-PARP, caspase 9, cleaved-caspase 9, caspase 3, cleaved-caspase 3, and GAPDH, diluted 1:1000 in Tris-buffered saline with Tween-20. Then, the membranes were incubated with peroxidase-conjugated secondary antibody diluted 1:2000. Finally, the protein bands on the membranes were scanned on an Odyssey CLx imaging system.

### *In vitro* cellular uptake analysis

Cellular uptake of PTX-ss-TMP NPs by A2780 cells was evaluated by FCM and confocal laser scanning microscopy (CLSM). PTX-ss-TMP NPs were labelled with Cy5 using a drug encapsulation approach, in which 0.1 mg/mL Cy5 was mixed with PTX-ss-TMP and dissolved in DMSO. A2780 cells were cultured in 12-well dishes (5 × 10^4^ cells/well) in DMEM medium containing 10% FBS overnight. Then, the cells were incubated with fresh medium without FBS containing 0.1 μg/mL free Cy5 or Cy5/PTX-ss-TMP NPs for 1, 2, or 4 h. Finally, the cells were collected and suspended in PBS. Intracellular Cy5 fluorescence was detected by FCM and analyzed with FlowJo software. Cells treated for 4 h were also washed thrice with cold PBS, fixed on cover slips with 4% paraformaldehyde, and double stained with Hoechst 33342 for nuclei and phalloidin for cytoskeleton. Then, the cells were imaged by CLSM (LEICA, Germany).

### Intracellular pharmacokinetics analysis

The concentrations of PTX and TMP in A2780 cells were determined by the ratio of intracellular drug concentration and amount of cell protein. A2780 cells were seeded in 6-well plates at a density of 4 × 10^5^ cells/well for 24 h. The cells were treated with PTX, TMP, or PTX-ss-TMP NPs (500 nM) for 0.5, 1, 2, and 4 h. Then, the cells were collected, washed twice with cold PBS, and resuspended in lysis buffer (800 μL) for 5 min. The cells were sonicated for 5 min to obtain cell lysate. Half of the cell lysate sample was removed to determine the protein amount using a BCA protein assay kit. The remaining half was mixed with 500 μL methyl *tert*-butyl ether to facilitate extraction of the drugs. Docetaxel (15 μL) was added to each sample as an internal standard. After vigorous mixing for 5 min, the samples were centrifuged at 13,000 rpm for 5 min. The intracellular concentrations of PTX and TMP were measured by HPLC.

### *In vitro* anti-angiogenesis assays

Cytotoxicity, cell proliferation, transwell migration, and tube formation assays were performed on HUVECs, as previously described 5. HUVECs were exposed to 0.1, 0.3, or 1 μM TMP, PTX, PTX+TMP mixture, or PTX-ss-TMP NPs for 48 h. Untreated cells were used as controls. Cytotoxicity and HUVEC viability were assessed by MTT and LDH assays, respectively. Proliferation was evaluated in cells stimulated by VEGF (50 ng/mL) and treated with 0.1 μM of the various treatments. Cell viability was evaluated as a percentage of the negative control without VEGF treatment. Each experiment was repeated at least three times independently.

Transwell migration and tube formation assays were performed using the non-cytotoxic dosage of 0.1 μM PTX. HUVECs were seeded in Transwell chambers and exposed to TMP, PTX, PTX+TMP mixture, or PTX-ss-TMP NPs. Cell medium containing 50 ng/mL VEGF was added to the lower compartment to stimulate cell migration. The cells were allowed to migrate for 2 h. Migrated cells were fixed with 4% paraformaldehyde, stained with 1:1000 (v/v) Hoechst 33342 for 30 min, photographed under a light microscope, and quantified by manual counting. For the tube formation assay, 24-well plates were coated with 300 μL Matrigel per well. HUVECs were seeded, treated with the various PTX and TMP treatments, and incubated for 4 h. The cells were imaged at 50× magnification using an inverted fluorescence microscope.

### *In vivo* biodistribution study

A2780 tumor-bearing nude mice were generated by subcutaneous injection of 0.2 mL A2780 cell suspension containing 5 × 10^6^ A2780 cells into the right flank of female BALB/c nude mice (5-7 weeks). Free Cy5 or Cy5-loaded PTX-ss-TMP NPs at a Cy5 dose of 0.1 mg/kg were injected into the tail vein. The fluorescence distribution was measured 24 h postinjection using an *in vivo* imaging system with excitation at 650 nm and emission collected at 670 nm (n = 3 at each time point). Tumor tissues and major organs were harvested at 24 h postinjection, rinsed with saline, and immediately imaged.

### *In vivo* therapeutic study

When the tumor volume reached ~250 mm^3^, A2780 tumor-bearing mice were intravenously administrated saline (control), TMP, PTX, PTX+TMP mixture, or PTX-ss-TMP NPs via the tail vein on day 0, 2, 4, 6, 8, and 10. The administered dosage was 10 mg/kg PTX or 1.6 mg/kg TMP according to the molar ratio of 1:1 in PTX-ss-TMP. Tumor size and body weight were monitored every 2 days for 12 days. Tumor volume was calculated as length × width^2^ / 2. On day 12, the animals were sacrificed and their tumors were dissected, photographed, and weighed to evaluate therapeutic efficacy. Then, tumor tissues from each group were fixed in formalin, embedded in paraffin, and stained with hematoxylin and eosin (H&E), *In Situ* Cell Death Detection Kit (Roche Applied Science, USA) for TUNEL assay, and anti-VEGF and anti-CD31 antibodies for immunohistochemistry 31. Major organs including heart, liver, spleen, lung, and kidney were also harvested for H&E staining to evaluate tissue toxicity by histopathological analysis.

### Statistical analysis

Data are expressed as mean ± SD and were evaluated using single-factor analysis of variance (ANOVA) followed by Student's *t*-test. Differences were considered statistically significant when p < 0.05 between groups.

## Results and Discussion

### Synthesis and characterization of PTX-ss-TMP conjugate

The amphiphilic redox-sensitive PTX-ss-TMP conjugate was synthesized by coupling TMP to PTX through a disulfide linkage via the multistep reaction shown in **Figure 1A**. ^1^H NMR, MALDI-TOF/MS, and UV-vis absorption analysis demonstrated the successful synthesis of PTX-ss-TMP. Both the intermediate and end product were determined by ^1^H NMR spectroscopy (**Figure 1B**). As shown, some representative chemical shift peaks of TMP and DTPA were found in the ^1^H NMR spectrum of TMP-DTPA, such as peak ***a*** at ~2.53 ppm that is related to (s, 3H, -CH_3_) on pyrazine and peak ***c*** at ~5.26 ppm that is related to (t, 2H, -CH_2_) on DTPA. Additionally, the main characteristic resonances of PTX and TMP-DTPA appeared on the ^1^H NMR spectrum of PTX-ss-TMP, demonstrating the successful conjugation of PTX to the carboxyl group of TMP-DTPA. Specifically, the characteristic resonance of the 2'-CH proton of free PTX was shifted from 4.69 ppm to 5.52 ppm in conjugated PTX. Additionally, the existence of peak 7-CH at 4.43 ppm in both free PTX and PTX-ss-TMP suggested that the esterification reaction took place preferentially at the 2'-hydroxyl of PTX due to the lower steric hindrance, which is in accordance with a previous publication 32.

The UV-vis absorption spectrum of PTX-ss-TMP (**Figure 1C**) was different from those of PTX and TMP. The absorption peak of TMP at 281 nm was blueshifted to 278 nm in PTX-ss-TMP, and the shoulder peak of PTX at 227 nm was weakened after conjugation with TMP. As shown in **Figure 1D**, generation of PTX-ss-TMP was also indicated by the peak at m/z 1180.4 (m/z, [M+H]^+^), which was in accordance with the theoretical molecular weight of 1179. Additionally, the structure of PTX-ss-TMP was identified by^ 13^C NMR spectroscopy (**Figure S1**). In summary, these results demonstrated the successful synthesis of PTX-ss-TMP.

### Preparation and characterization of PTX-ss-TMP NPs

**Figure 2:**
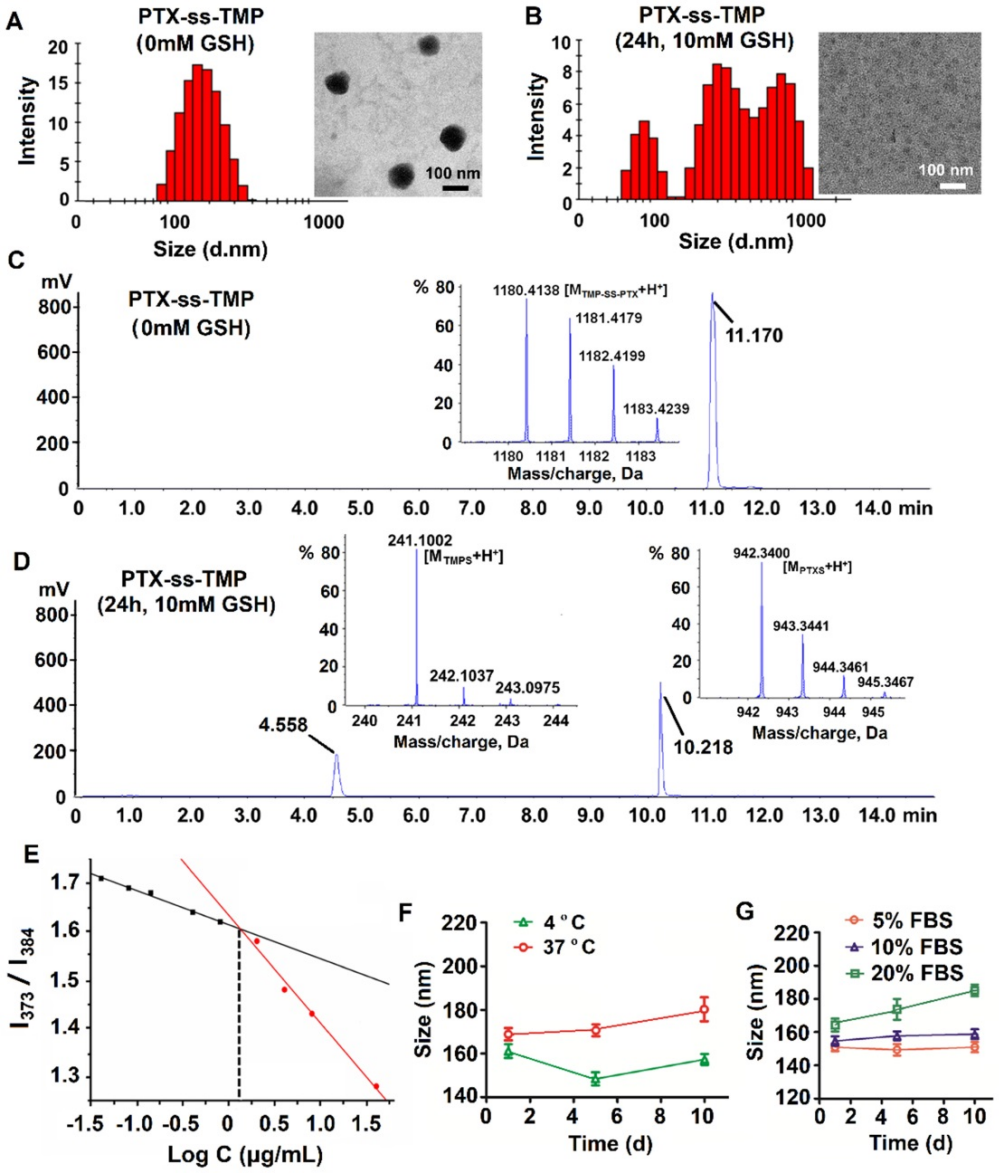
** Characterization of PTX-ss-TMP NPs. (A-B)** Particle size distribution by DLS and TEM images of PTX-ss-TMP NPs in the absence (A) and presence (B) of 10 mM GSH. **(C-D)** HPLC-TOF/MS analysis of PTX-ss-TMP NPs in the absence (C) and presence (D) of 10 mM GSH. **(E)** CAC determination of PTX-ss-TMP using the probe pyrene. **(F-G)** Particle size by DLS of PTX-ss-TMP NPs incubated for 10 days in PBS (F) and cell culture medium containing FBS (G).

The amphiphilic drug conjugate PTX-ss-TMP was self-assembled in aqueous solution via a nanoprecipitation approach. For this process, organic phases including methanol, acetone, and DMSO were screened based on the resulting particle distribution and drug encapsulation. PTX-ss-TMP NPs formed with DMSO were ~152 nm in size and had a spherical morphology (**Figure 2A**), whereas PTX-ss-TMP NPs formed with either methanol or acetone were larger than 300 nm. The zeta potential of PTX-ss-TMP NPs was -23.3 ± 2.8 mV, indicating that the NPs should have good stability, blood compatibility, and prolonged circulation time due to reduced interactions with blood components compared with non-negatively charged NPs.

### Redox sensitivity of PTX-ss-TMP NPs

The redox sensitivity of PTX-ss-TMP NPs was evaluated by changes in their size distribution, TEM morphology, and chromatography/mass spectrometry profiles under reductive conditions. As is well known, disulfide bonds are fairly stable in normal biological conditions but are rapidly cleaved in tumors through thiol-disulfide exchange reactions facilitated by high intracellular concentrations of GSH. The cleavage of disulfide bond into thiol in the response to 10 mM GSH have been occurred in some previous redox-sensitive drug-drug conjugate 33, 34. After treated with 10 mM GSH for 24 h, with the cleavage of the disulfide linkage in drug conjugate, dramatic fracture and aggregation was occurred 28. Under these conditions, PTX-SS-TMP NPs also disintegrated, their size distribution broadened, and their spherical morphology was lost, as shown in **Figure 2B**. Some PTX precipitated in the aqueous phase immediately and formed needle-like crystals. This result might be due to redox-triggered cleavage of the disulfide bonds and subsequent release of hydrophobic PTX-SH, which aggregated into macroparticles in the water 35. And compared to the non-significant changes of PTX-ss-TMP NPs without GSH treatment, the spherical morphology of PTX-ss-TMP NPs was disappeared (**Figure 2B**). To further corroborate the redox sensitivity of PTX-ss-TMP NPs, the chromatography and mass spectrometry profiles of PTX-ss-TMP NPs with or without GSH treatment were analyzed by HPLC-TOF/MS. The retention time of PTX-ss-TMP was 11.17 min in the absence of GSH (**Figure 2C**), whereas only free thiolated TMP (4.558 min) and free thiolated PTX (10.218 min) were detected in the presence of 10 mM GSH (**Figure 2D**). The reduction-responsive drug release behavior of PTX-ss-TMP NPs was further evaluated using a dialysis method. As shown in **Figure S3**, the drug release profiles of both PTX and TMP from PTX-ss-TMP NPs in 10 µM GSH were relatively slow over 72 h, whereas drug release was dramatically enhanced in 10 mM GSH. The mechanisms underlying the redox-responsive drug release of PTX-ss-TMP triggered by GSH are shown in **Figure S4**. The disulfide bond in PTX-ss-TMP was degraded into thiol groups in the presence of GSH, and the generated hydrophilic thiol groups could facilitate hydrolysis of the adjacent ester bond and release PTX from the prodrug. Therefore, free PTX and 2-hydroxylmethyl-TMP molecules would be released during exposure to GSH, instead of free thiolated PTX or free thiolated TMP. 2-hydroxylmethyl-TMP is the derivative product of TMP in the synthesis of PTX-ss-TMP, as well as the metabolite of TMP *in vivo*. Both TMP and the generated 2-hydroxylmethyl-TMP possess the same nuclear parent and have similar therapeutic effects. As most solid tumors are characterized by high GSH concentration, these results suggest that PTX-ss-TMP NPs could rapidly break down and release drugs into the tumor microenvironment after accumulating in the tumor by the enhanced permeability and retention (EPR) effect, leading to enhanced antitumor efficacy.

### CAC, hemolytic effect, and colloidal stability of PTX-ss-TMP NPs

The CAC of PTX-ss-TMP was measured using the probe pyrene to investigate the self-assembly behavior of PTX-ss-TMP NPs. The relationship between pyrene fluorescence intensity I_373_/I_384_ ratio and PTX-ss-TMP concentration is presented in **Figure 2E**. According to the inflection point of the curve, the CAC value of PTX-ss-TMP was 1.35 μg/mL, which is much lower than that of an irinotecan-chlorambucil conjugate previously published 26.

The hemolytic effect of PTX-ss-TMP NPs was evaluated using rabbit erythrocytes. As shown in **Figure S5**, 0.5 mg/mL PTX-ss-TMP NPs showed negligible hemolysis. The hemolytic rate was determined to be only 1.34% according to hemoglobin absorbance, which is less than the upper limit of 5% for systemic administration with low toxic effects 36.

The colloidal stability of PTX-ss-TMP NPs was assessed in PBS (pH 7.4) and RPMI 1640 media containing 5%, 10%, or 20% FBS by DLS. PTX-ss-TMP NPs exhibited no significant change in particle size over 10 days storage in PBS at both 4 and 37 ºC (**Figure 2F**). Additionally, 5-20% FBS did not dramatically increase the particle size (**Figure 2G**), potentially due to weak protein adsorption. These results indicated the good *in vitro* storage stability of PTX-ss-TMP NPs. Furthermore, the solubilization effect of PTX-ss-TMP is presented in **Figure S6**. PTX-ss-TMP NPs exhibited homogeneous and stable dispersions with blue opalescence in comparison to the rapid precipitation of PTX and slight dissolution of TMP.

### Cytotoxicity of PTX-ss-TMP NPs against ovarian cancer cells

**Figure 3:**
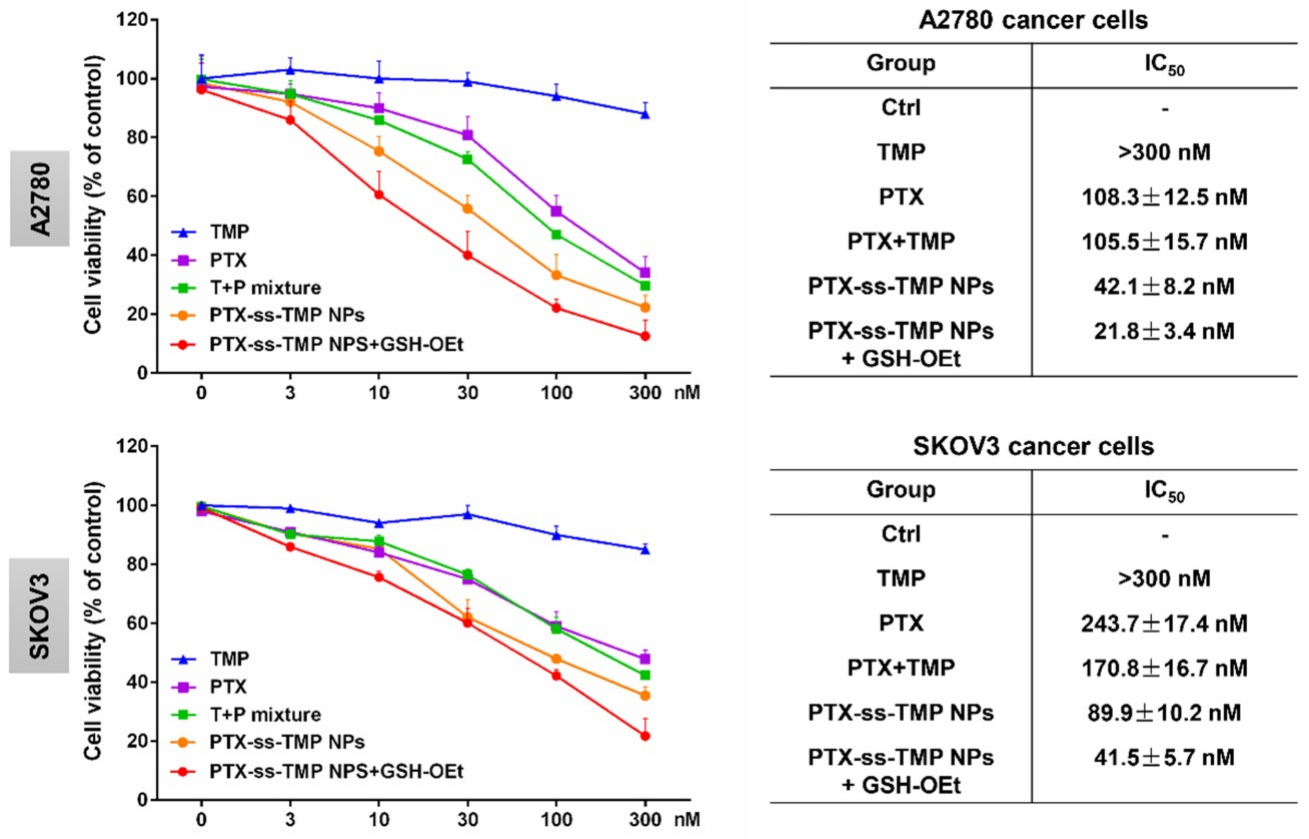
***In vitro* cytotoxicity of PTX-ss-TMP NPs against ovarian cancer cells.** A2780 and SKOV3 cells were incubated with free TMP, free PTX, PTX+TMP mixture, and PTX-ss-TMP NPs for 48 h and then cell viability was measured by MTT assay. Pretreatment with GSH-OEt for 2 h was employed to increase the intracellular GSH concentration through ethyl ester hydrolysis in the cytoplasm.

A2780 and SKOV3 ovarian cancer cells were employed to evaluate the cytotoxicity of PTX-ss-TMP NPs. Cells were incubated with various PTX and TMP treatments for 48 h and their viability was measured by MTT assay. As shown in **Figure 3**, in contrast to the non-significant cytotoxicity of free TMP, the treatments containing PTX exhibited obvious dose-dependent suppression of cell proliferation. Remarkably inhibited cell proliferation was observed with PTX-ss-TMP NPs compared with free PTX and PTX+TMP mixture. Addition of GSH-OEt to culture medium has been reported to rapidly increase the intracellular concentration of GSH through ethyl ester hydrolysis in the cytoplasm 37. Pretreatment of the ovarian cancer cells with GSH-OEt for 2 h significantly enhanced the cytotoxicity of PTX-ss-TMP NPs. The average IC_50_ values were 42.1 nM and 21.8 nM in A2780 cells for PTX-ss-TMP NPs without and with GSH-OEt pretreatment, respectively. Similarly, GSH-OEt pretreatment of SKOV3 cells almost halved the IC_50_ value of PTX-ss-TMP NPs. This difference may result from rapid cleavage of disulfide bonds in PTX-ss-TMP NPs and subsequent drug release mediated by the increased intracellular reducibility. These results suggested that self-assembly of PTX-ss-TMP into NPs increased the cytotoxicity of the combination PTX and TMP treatment, and cleavage of the disulfide bonds increased the cytotoxicity of the NPs against cancer cells.

### Apoptosis induction by PTX-ss-TMP NPs in ovarian cancer cells

**Figure 4:**
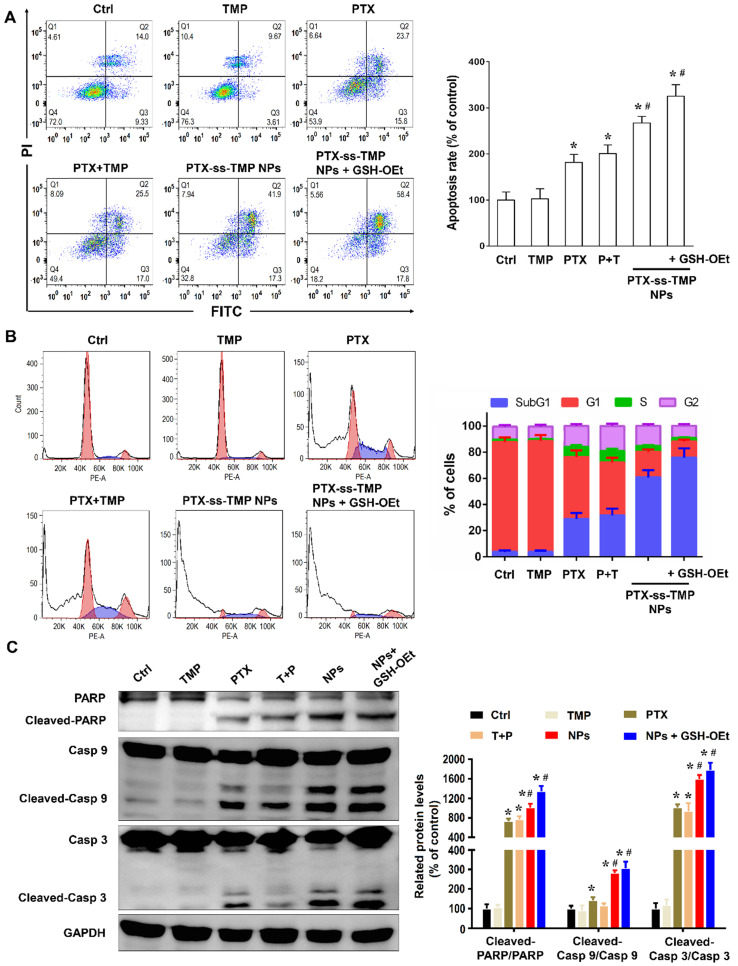
** Apoptosis induction by PTX-ss-TMP NPs *in vitro*.** A2780 cells were incubated with 100 nM free TMP, free PTX, PTX+TMP mixture, and PTX-ss-TMP NPs for 48 h and then analyzed. Pretreatment with GSH-OEt for 2 h was employed to increase the intracellular GSH concentration through ethyl ester hydrolysis in the cytoplasm.** (A)** Apoptotic cells detected by FCM with Annexin V-FITC/PI double staining. **(B)** Cell cycle analysis by FCM. G0/G1, G2/M, and S indicate cell phases, and sub-G0/G1 refers to the proportion of apoptotic cells. **(C)** Expression levels of apoptosis-related proteins. *P < 0.05 vs. untreated control, ^#^P < 0.05 vs. PTX group.

As one of the most important mechanisms inducing cell death, apoptosis was evaluated in A2780 cells treated with various PTX and TMP treatments for 48 h. Treated cells were double stained with Annexin V-FITC/PI and then analyzed by FCM. As shown in **Figure 4A**, the apoptosis rates (calculated by adding the upper-right and lower-right quadrant values) induced by the various PTX treatments were in accordance with the cytotoxicity results, in which PTX-ss-TMP NPs exhibited a much higher apoptosis rate than both free PTX and PTX+TMP mixture. In particular, pretreatment with GSH-OEt for 2 h significantly increased the apoptosis rate induced by PTX-ss-TMP NPs from 59.2% to 76.2%.

As is well known, PTX binds to α, β-tubulin dimers within microtubules, thereby inducing G2/M phase arrest and proliferation inhibition. **Figure 4B** shows that cells treated with free PTX or PTX+TMP mixture were arrested in the G2/M phase with considerably reduced accumulation in the G1 phase. Interestingly, the percentage of cells in the subG1 phase was increased by PTX-ss-TMP NPs treatment with or without OEt induction, which resulted in enhanced apoptosis. This result was consistent with the enhanced apoptosis rate caused by PTX-ss-TMP NPs treatment in **Figure 4A**.

We further evaluated the effects of PTX-ss-TMP NPs on the expressions of some apoptosis-related proteins, including the cleaved and total amounts of PARP, caspase 9, and caspase 3. All these proteins have been revealed to be involved in PTX-induced apoptosis. As shown in **Figure 4C**, free PTX greatly increased the ratio of cleaved to total protein. However, treatment with PTX-ss-TMP NPs significantly further enhanced PTX activity on PARP, caspase 9, and caspase 3. Likewise, pretreatment with GSH-OEt further heightened the expressions of apoptosis-related proteins induced by PTX-ss-TMP NPs, which was in accordance with the cytotoxicity and apoptosis results from FCM. These results corroborated the enhanced cell apoptosis effect of PTX-ss-TMP NPs, especially in response to high intracellular reducibility.

### Cellular uptake of PTX-ss-TMP NPs

**Figure 5:**
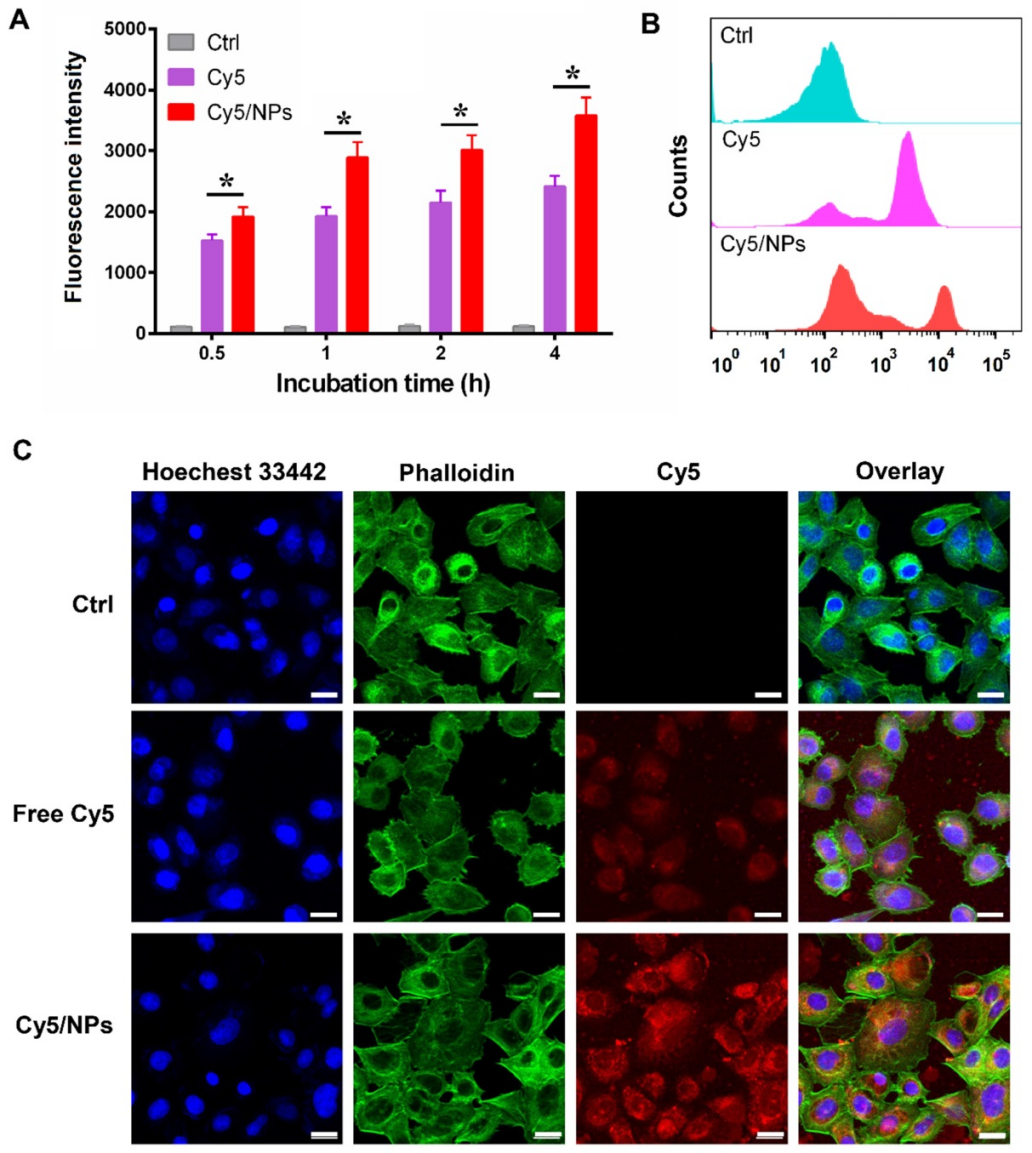
** Cellular uptake of Cy5-labeled PTX-ss-TMP NPs by A2780 cells. (A)** Time-dependent fluorescence intensity by FCM. **(B)** Representative FCM spectra. **(C)** Representative CLSM images after 4 h incubation. Cell nuclei and cytoskeleton were stained with Hoechst 33342 and phalloidin, respectively. Scale bars are 20 μm.

To investigate our assumption that the increased cytotoxicity of PTX-ss-TMP NPs compared with free drugs resulted from increased cell uptake of the nanoparticles, the near-infrared fluorophore Cy5 was encapsulated into PTX-ss-TMP NPs using a similar nano-assembly approach as that described in the PTX-ss-TMP NPs preparation section. We evaluated leakage of Cy5 from PTX-ss-TMP NPs in cell culture medium by dialysis. After 1, 2, and 4 h incubation, the release rates of Cy5 were 3.1%, 6.5%, and 8.3%, respectively. Therefore, we deemed that Cy5 loading in PTX-ss-TMP NPs was sufficiently stable for cellular uptake experiments. Cellular uptake of free Cy5 and Cy5/PTX-ss-TMP NPs by A2780 cells was investigated by FCM (**Figure 5A-B**). A time-dependent increase in Cy5 fluorescence was observed for both free Cy5 and Cy5/PTX-ss-TMP NPs. However, cells treated with Cy5/PTX-ss-TMP NPs had much stronger fluorescence intensities than those treated with free Cy5 at each time point, indicating that PTX-ss-TMP NPs possessed a higher cellular uptake capacity compared with free drugs.

We corroborated these FCM results with CLSM to assess subcellular localization. A2780 cells were treated with free Cy5 or Cy5/PTX-ss-TMP NPs for 4 h, then the nuclei and cytoskeleton were stained with Hoechst 33342 and phalloidin, respectively. As shown in **Figure 5C**, much stronger Cy5 fluorescence was observed in the cytoplasm of cells treated with Cy5/PTX-ss-TMP NPs than free Cy5, indicating higher cellular uptake. Furthermore, we also directly determined the intracellular concentrations of PTX and TMP in A2780 cells after treatment with free TMP, free PTX, or PTX-ss-TMP NPs. As shown in **Figure S7**, intracellular concentrations of PTX and TMP from PTX-ss-TMP NPs were markedly higher than those from free drugs. PTX-ss-TMP NPs exhibited 3.68-, 3.04-, 3.66-, and 2.69- fold higher accumulation in the cytoplasm of A2780 cells than free PTX after 0.5, 1, 2, and 4 h of incubation, respectively. This result was consistent with the characterization analysis using the fluorophore Cy5. In summary, the above results suggested that PTX-ss-TMP NPs had higher cellular uptake, more efficient cytoplasmic accumulation of drugs, and enhanced cancer cell killing than free drugs.

### Effect of PTX-ss-TMP NPs on angiogenesis in HUVECs

**Figure 6:**
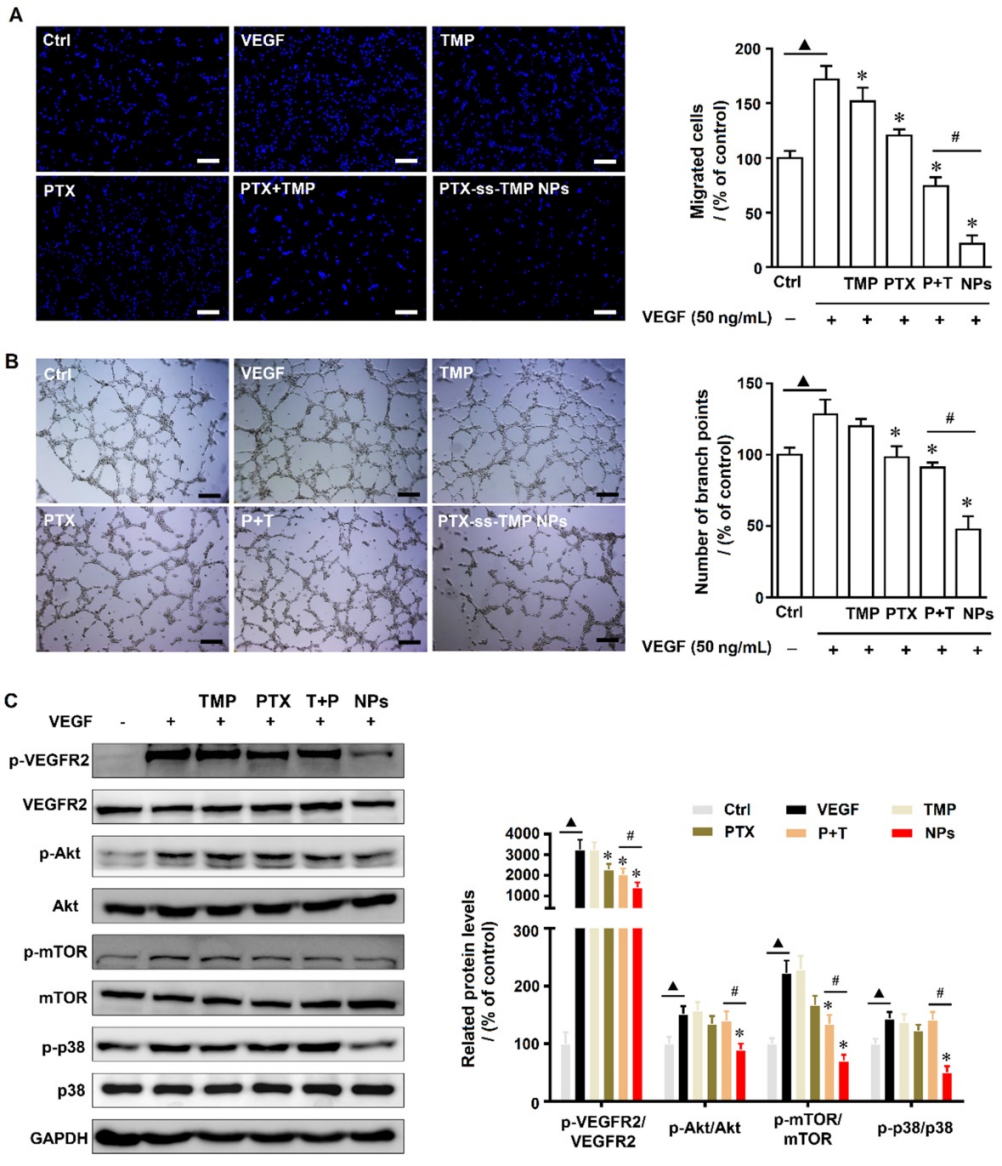
**Effects of PTX-ss-TMP NPs on angiogenesis* in vitro*.** HUVECs were pretreated with VEGF and then treated with 0.1 μM free TMP, free PTX, PTX+TMP mixture, and PTX-ss-TMP NPs for 2 h. Transwell migration **(A)** and tube formation** (B)** of HUVECs on Matrigel. **(C)** Expression levels of VEGFR-related pathways including phosphorylated and unphosphorylated VEGFR2, Akt, mTOR, and P38. ^▲^P < 0.05 vs. untreated control, *P < 0.05 vs. VEGF group, ^#^P < 0.05 vs. PTX+TMP mixture group. Scale bars are 20 μm.

Angiogenesis is a physiologically complex process involving the proliferation and migration of endothelial cells that is suppressed by both PTX 38 and TMP 39. We evaluated the angiogenesis suppression effects of PTX-ss-TMP NPs by endothelial cell proliferation, migration, and tube formation assays. First, a non-cytotoxic dosage of PTX-ss-TMP NPs was determined by MTT and LDH assays. As shown in **Figure S8**, 0.3 and 1 μM PTX-ss-TMP NPs inhibited HUVEC viability after 48 h incubation, whereas 0.1 μM did not significantly impact viability. Additionally, increased LDH release in culture medium was observed at the 1 μM dosage, indicating potential cellular damage. Thus, we chose 0.1 μM as a safe dosage for subsequent experiments. Significant growth of HUVECs was induced by 50 ng/mL VEGF. Treatment with 0.1 μM PTX-ss-TMP NPs for 4 h impaired this induced cell growth more than the other treatments. Furthermore, while all PTX treatments suppressed VEGF-enhanced HUVEC migration and tube formation, PTX-ss-TMP NPs caused the strongest decrease in migrated cells and formed branch points (**Figure 6A-B**). These results indicated that PTX-ss-TMP NPs had significantly enhanced anti-angiogenesis effects than either free PTX or PTX+TMP mixture.

The anti-angiogenesis mechanisms were evaluated by western blotting of angiogenesis-involved proteins. VEGFR-2 plays an important role in mediating the mitogenesis and permeability of endothelial cells, and has become an important therapeutic target for anti-angiogenesis cancer therapy 40. Activation of VEGFR-2 results in phosphorylation of multiple downstream signals including Akt, Erk, and JNK. Additionally, phosphorylation of p38 and mTOR kinase signaling pathways is involved in the proliferation and migration of endothelial cells. **Figure 6C** demonstrates that PTX-ss-TMP NPs significantly suppressed the VEGFR2-AKT/mTOR/p38 signaling axis and remarkably decreased the expression levels of p-VEGFR2/VEGFR2, p-AKT/AKT, p-mTOR/mTOR, and p-p38 /p38. These results provided clear evidence that PTX-ss-TMP NPs suppressed HUVEC angiogenesis via VEGFR2 and involved downstream signals.

### *In vivo* biodistribution of PTX-ss-TMP NPs

**Figure 7:**
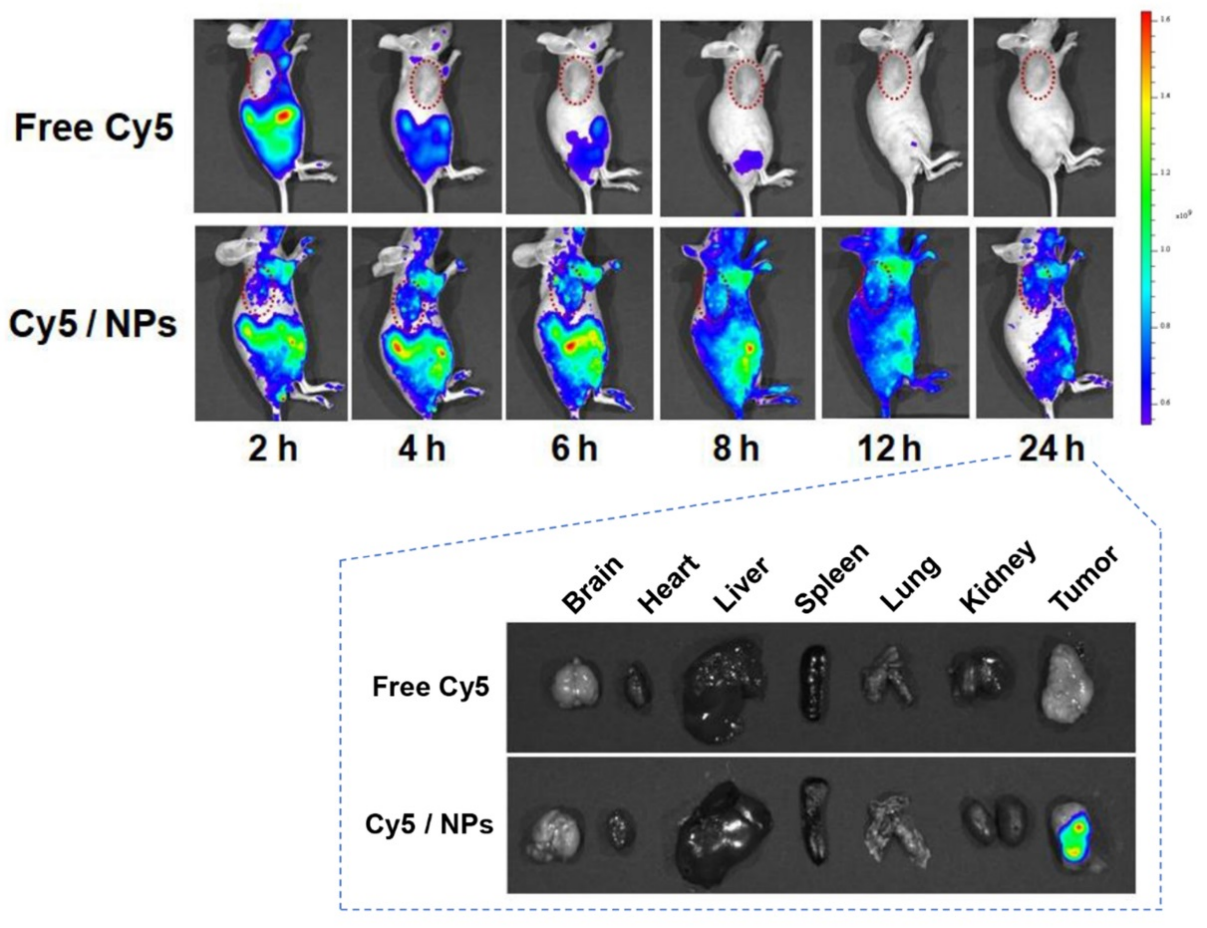
**Biodistribution of Cy5/PTX-ss-TMP NPs.** A2780 tumor-bearing mice were intravenously administered free Cy5 or Cy5/PTX-ss-TMP NPs and imaged for 24 h. Tumors and major organs were excised at 24 h for *ex vivo* imaging. Tumors are indicated by red circles.

It is well known that nanosystems of a suitable size usually display longer retention and better accumulation in tumors than small molecules due to the EPR effect. We used* in vivo* fluorescence imaging to preliminarily characterize the biodistribution of PTX-ss-TMP NPs in mice bearing A2780 tumor xenografts (**Figure 7**). Compared to the rapid clearance and poor tumor accumulation of free Cy5, Cy5-loaded PTX-ss-TMP NPs exhibited efficient tumor accumulation over 24 h after intravenous injection. The tumor and major organs were also excised for *ex vivo* fluorescence imaging at 24 h postinjection. The tumors of mice administered Cy5/PTX-ss-TMP NPs showed significantly stronger fluorescence than those administered free Cy5. There was no obvious retention of fluorescence signal in major organs, indicating the ideal tumor accumulation of PTX-ss-TMP NPs.

### *In vivo* antitumor efficacy of PTX-ss-TMP NPs

**Figure 8:**
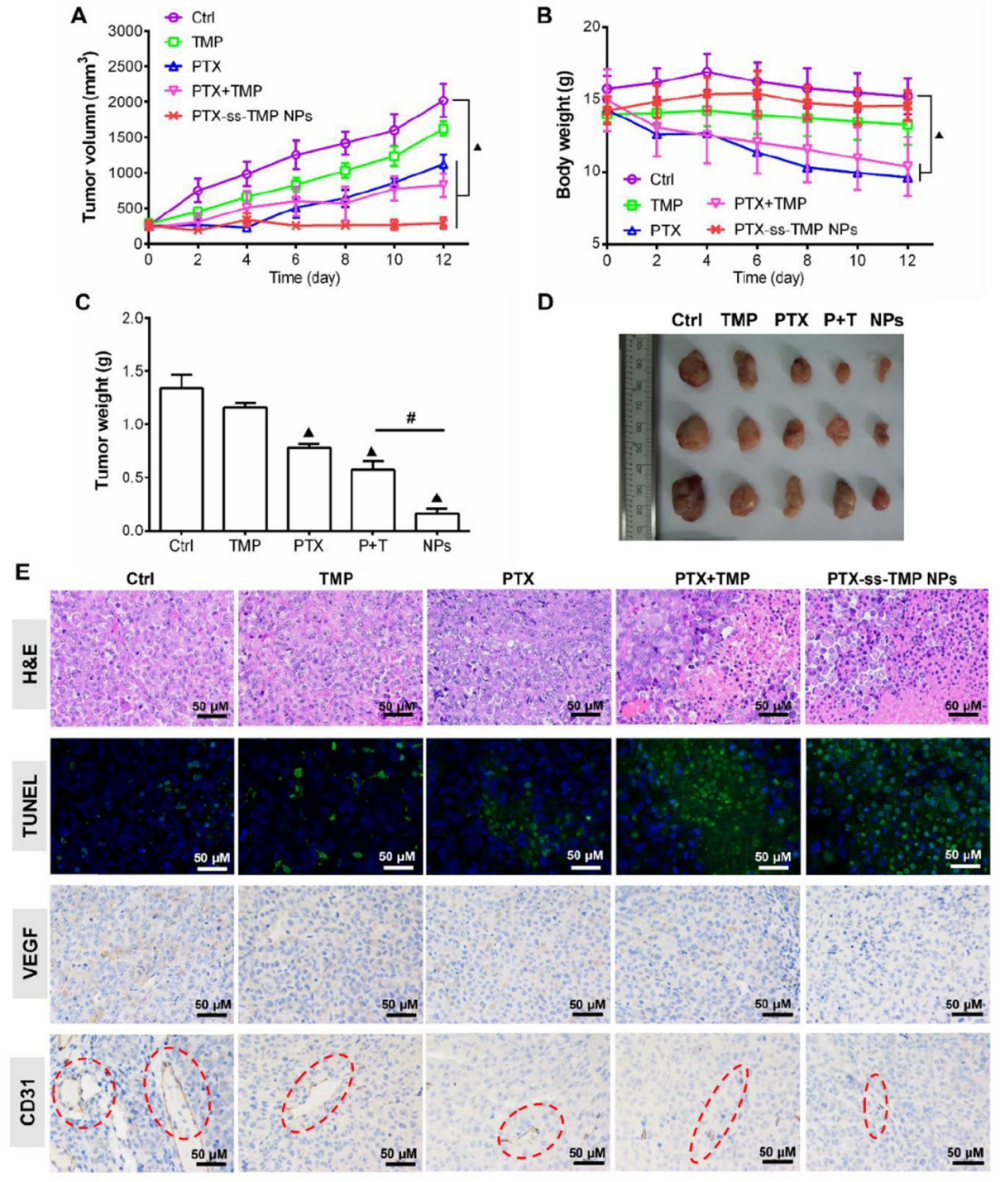
***In vivo* antitumor effect of PTX-ss-TMP NPs in A2780 tumor-bearing mice.** Mice were intravenously injected every 2 days with saline, TMP (1.6 mg/kg), PTX (10 mg/kg), PTX+TMP mixture (11.6 mg/kg), or PTX-ss-TMP NPs (12 mg/kg) at equivalent doses of TMP and/or PTX for 12 days.** (A)** Tumor volume changes. **(B)** Body weight changes.** (C)** Excised tumor weight.** (D)** Representative images of excised tumors.** (E)** Tumor tissue stained for H&E, TUNEL, VEGF, and CD31. Red circles highlight microvessels. ^▲^P < 0.05 vs. saline control, ^#^P < 0.05 NPs vs. PTX+TMP mixture group.

The antitumor effect of PTX-ss-TMP NPs was evaluated in female nude mice bearing A2780 tumor xenografts and compared to free TMP, free PTX, and their mixture. As shown in **Figure 8A**, all treatments inhibited tumor growth compared to saline control during the whole experimental period. Although free PTX and PTX+TMP mixture significantly suppressed tumor growth, systemic toxicity was also obvious (**Figure 8B**). These treatments resulted in substantial loss of body weight due to the lack of tumor targeting and systemic distribution of free PTX. In comparison, PTX-ss-TMP NPs showed higher antitumor efficacy and lower systemic toxicity, which was consistent with the results of the *in vitro* cytotoxicity assay. As shown in **Figure 8C**-**D**, the tumors of mice treated with PTX-ss-TMP NPs were much smaller than those of other treatment groups. The outstanding antitumor efficacy of PTX-ss-TMP NPs is hypothesized to result from three aspects: (i) the prolonged circulation, enhanced tumor accumulation, and increased cellular uptake of nanoparticles compared with free drugs; (ii) the expected high intracellular drug concentrations resulting from rapid NPs dissociation in response to GSH. (iii) the synergetic cancer cell apoptosis and anti-angiogenesis effects of the combination treatment. PTX-ss-TMP NPs were confirmed to cause both apoptosis and anti-angiogenesis effects by immunohistochemistry (**Figure 8E**). Tumor tissues harvested from mice treated with PTX-ss-TMP NPs exhibited less cell proliferation, more cell apoptosis, and fewer new blood vessels compared to the other treatment groups. Major organs including heart, liver, spleen, lung, and kidney were also collected for H&E examination. Although significant loss in body weight was observed in mice treated with free PTX and PTX+TMP mixture, only free PTX caused slight damage to cardiac muscle (**Figure S9**). PTX-ss-TMP NPs did not cause obvious histopathological changes to major organs. Therefore, these results demonstrate that PTX-ss-TMP NPs are highly efficient and safe therapeutic agents for tumor therapy via cancer cell apoptosis induction and angiogenesis suppression.

## Conclusion

Synergistic chemotherapy with PTX and TMP is limited due to their asynchronous delivery to tumors. However, nanocarriers such as polymer nanoparticles have disadvantages including low drug loading and poor quality control. To address these challenges, we synthesized an amphiphilic redox-sensitive PTX-ss-TMP conjugate and assembled it into nanoparticles. PTX-ss-TMP NPs improved the cellular uptake and synchronized the intracellular delivery of PTX and TMP compared with free drugs, resulting in highly efficient cytotoxicity against A2780 and SKOV3 ovarian cancer cells. Pretreatment of A2780 cells with GSH-OEt to generate a high intracellular GSH concentration remarkably enhanced the cytotoxicity of PTX-ss-TMP NPs and increased the rate of apoptosis due to redox-responsive cleavage of PTX-ss-TMP. Furthermore, PTX-ss-TMP NPs combined the pro-apoptosis and angiogenesis inhibition effects of PTX and TMP. We evaluated the anti-angiogenesis efficacy of PTX-ss-TMP NPs in HUVECs and showed that the nanoparticle delivery strategy improved suppression of cell growth, migration, and tube formation mediated by inhibition of VEGF-involved pathways. Furthermore, as assessed by fluorescence imaging of loaded Cy5, PTX-ss-TMP NPs showed highly efficient tumor accumulation by EPR effect-mediated passive targeting. Finally, the combined suppression of cancer cells and new blood vessels resulted in superior synergistic treatment of A2780 tumor-bearing mice with reduced toxicity compared to monotherapy and combination therapy with free drugs. To sum up, this “all-in-one” nanosystem composed of drug-drug conjugates with tumor microenvironment-responsive linkages provides inspiration for maximizing the synergistic efficacy of combination chemotherapies.

## Supplementary Material

Supplementary figures and tables.
