# Supplementary material for: Redox-sensitive carrier-free nanoparticles self-assembled by disulfide-linked paclitaxel-tetramethylpyrazine conjugate for combination cancer chemotherapy
Source: Theranostics. 2021 Feb 20;11(9):4171–86. doi: 10.7150/thno.42260 (PMC7977472; doi:10.7150/thno.42260)
Supplement: Supplementary file 1 — Supplementary figures and tables. [file thnov11p4171s1.pdf]

Supporting Information

Redox-sensitive Carrier-free Nanoparticles self-assembled by Disulfide-linked Paclitaxel-Tetramethylpyrazine Conjugate for Combination Cancer Chemotherapy

Liang Zou[1,2], Xiaowei Liu[3], Jingjing Li[2,4], Wei Li[3], Lele Zhang[3], Chaomei Fu[2], Jinming Zhang[2*], Zhongwei Gu[5*]

1. Key Laboratory of Coarse Cereal Processing of Ministry of Agriculture and Rural Affairs, Chengdu University, Chengdu 610106, People's Republic of China
2. State Key Laboratory of Southwestern Chinese Medicine Resources, College of Pharmacy, Chengdu University of Traditional Chinese Medicine, Chengdu 611137, People's Republic of China
3. School of Basic Medical Sciences, Chengdu University, Chengdu 610106, People's Republic of China
4. Department of Pharmacology and Pharmacy, University of Hong Kong, Hong Kong 999077, People's Republic of China
5. College of Materials Science and Engineering, Nanjing Tech University, Nanjing 211816, People's Republic of China

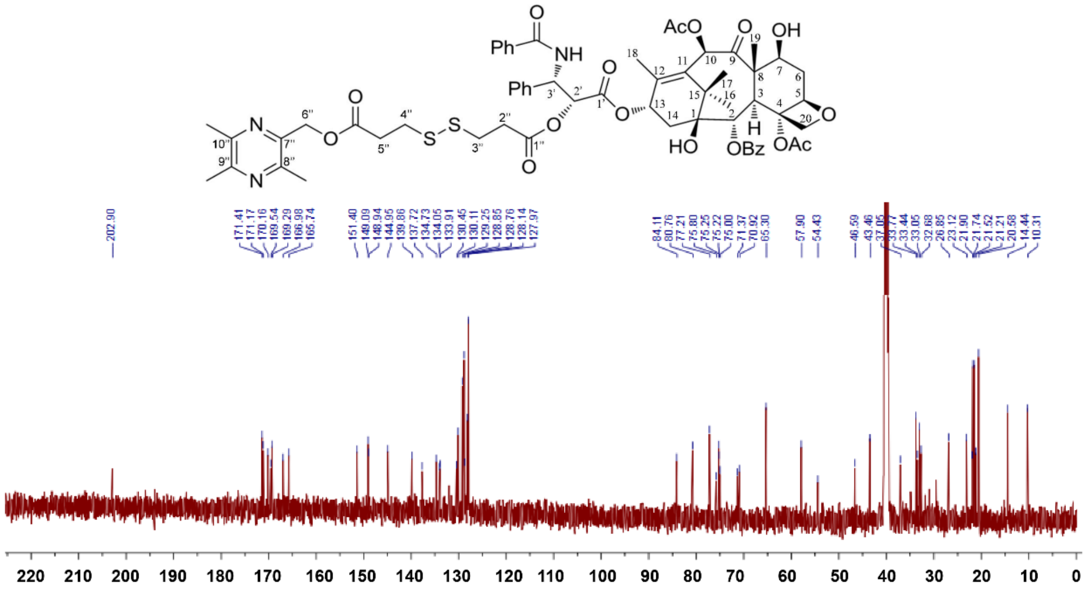

Figure S1. ^{13}C NMR spectrum of PTX-ss-TMP conjugate.

Table S1. ^{13}C NMR data of PTX-ss-TMP conjugate in DMSO-d_6 (150 MHz).

No.	δ (ppm)	No.	δ(ppm)	No.	δ(ppm)
1	77.2	15	43.3	6”	169.3
2	75.0	16	21.9	7”	151.4
3	46.6	17	26.9	8”	144.0
4	80.7	18	14.4	9”	148.9
5	84.1	19	10.31	10”	149.1
6	33.7	20	75.8	4-OAc	170.2, 23,2
7	70.9	1’	171.4	10-OAc	171.2, 21.7
8	57.9	2’	75.2	2-benzoyl	166.9, 130.1, 130.1, 127.9, 127.9, 133.9
9	202.9	3’	54.4	3’-N-benzoyl	169.5, 128.2, 128.2, 128.9, 128.9, 130.4
10	75.2	1”	165.7	3’-Ph	130.7, 127.9, 127.9, 129.3, 129.3, 128.8
11	134.0	2”	33.4	8”-Me	21.5
12	139.8	3”	65.3	9”-Me	20.6
13	71.4	4”	65.3	10”-Me	21.2
14	37.0	5”	33.1		

ESI spectrum of TMP-ss-PTX conjugate

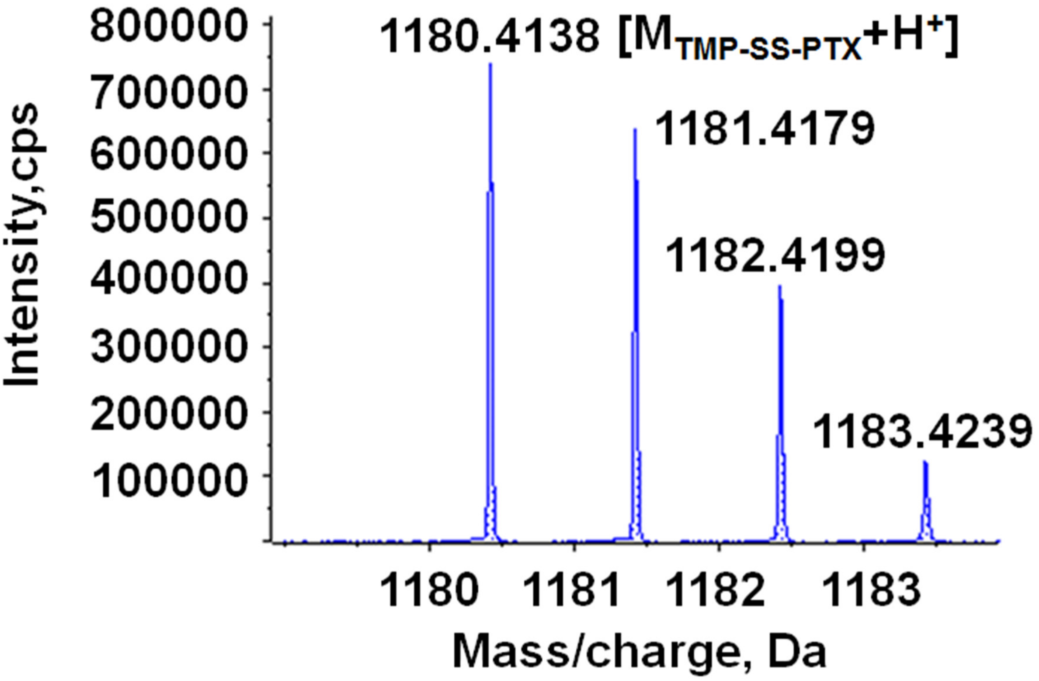

Figure S2. Q-TOF MS/MS spectrum of PTX-ss-TMP conjugate.

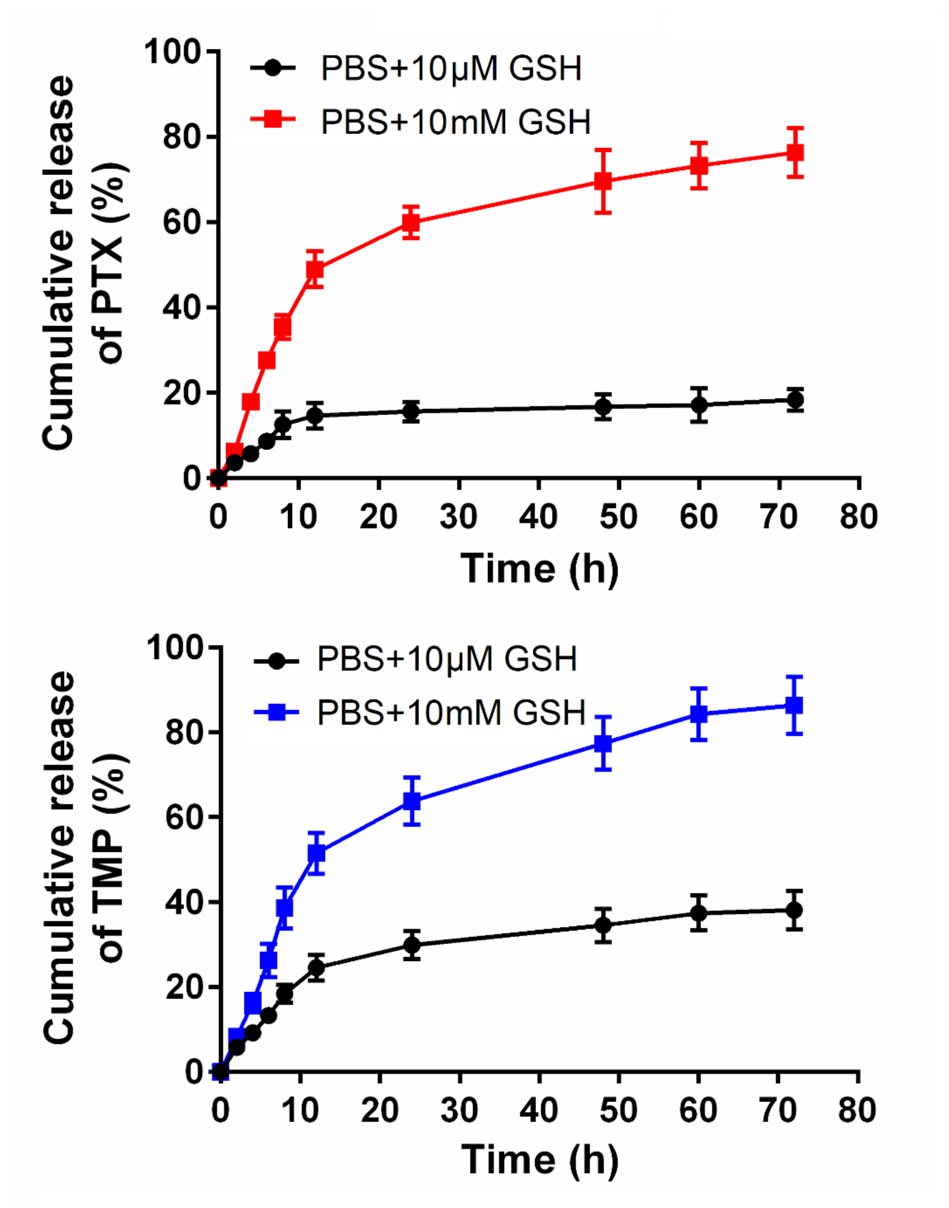

Figure S3. Redox-triggered drug release from the PTX-ss-TMP NPs at 37 °C in PBS with or without 10nM GSH (pH 7.4, n = 3).

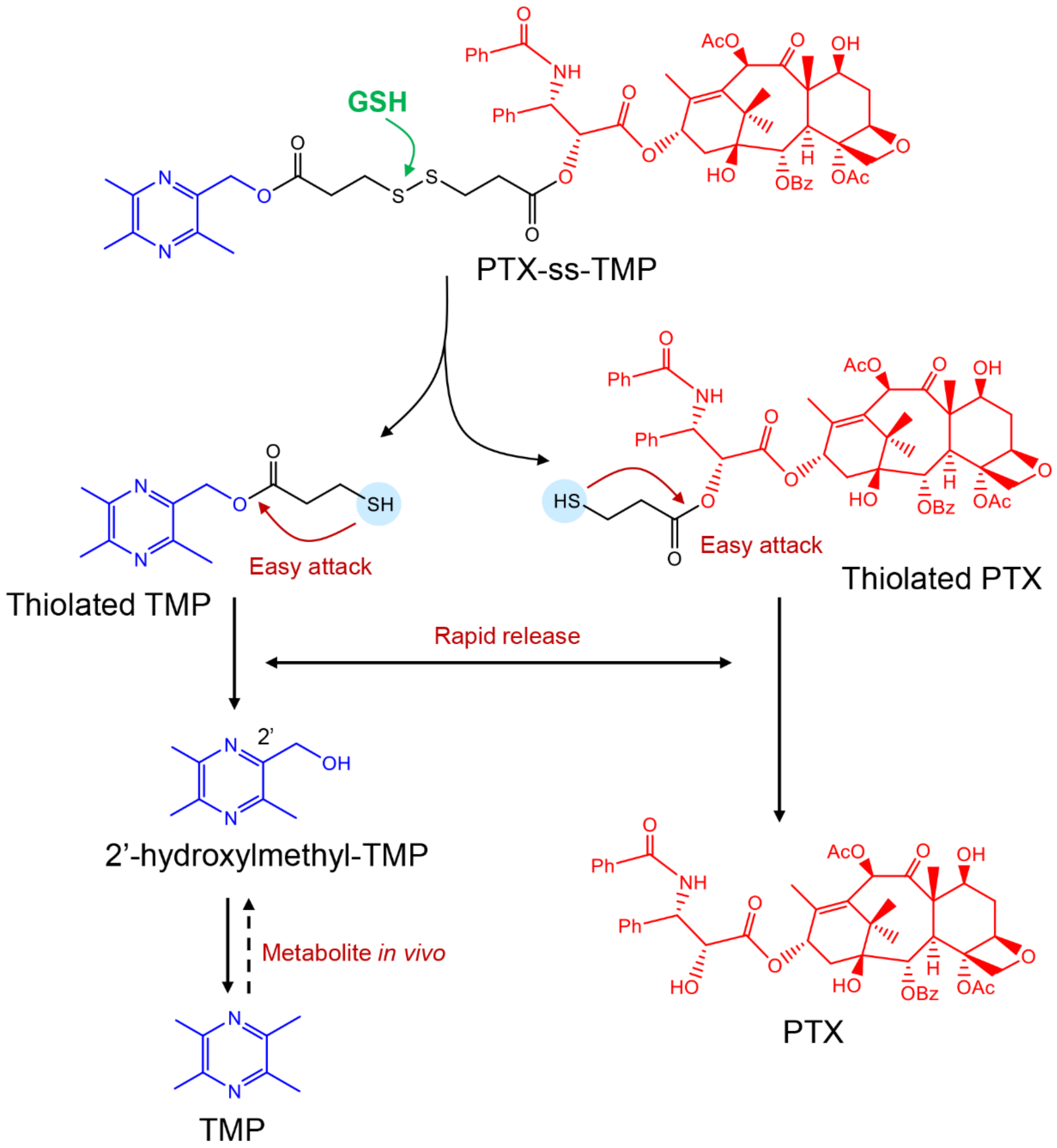

Figure S4. Mechanisms of the redox responsive drug release of disulfide bond triggered by GSH.

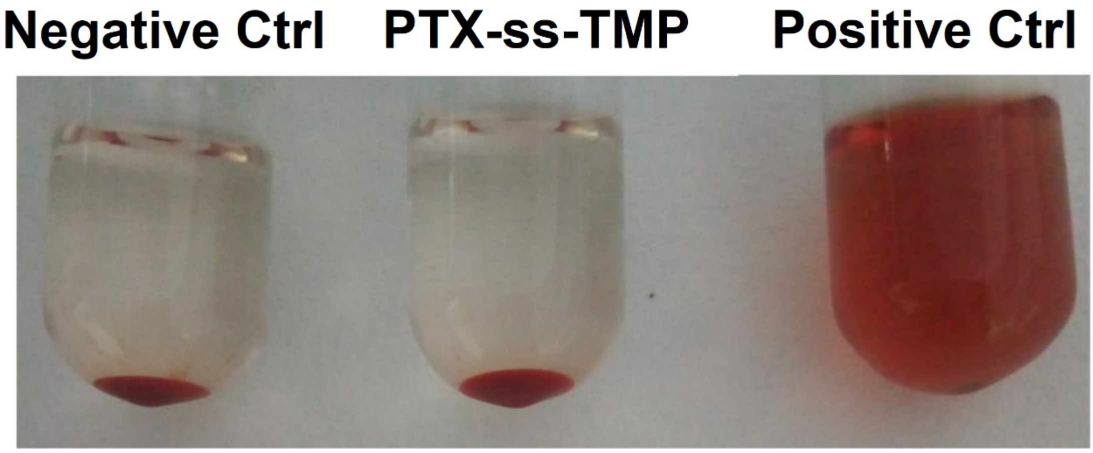

Figure S5. Hemolysis results of PTX-ss-TMP NPs after 24 h incubation.

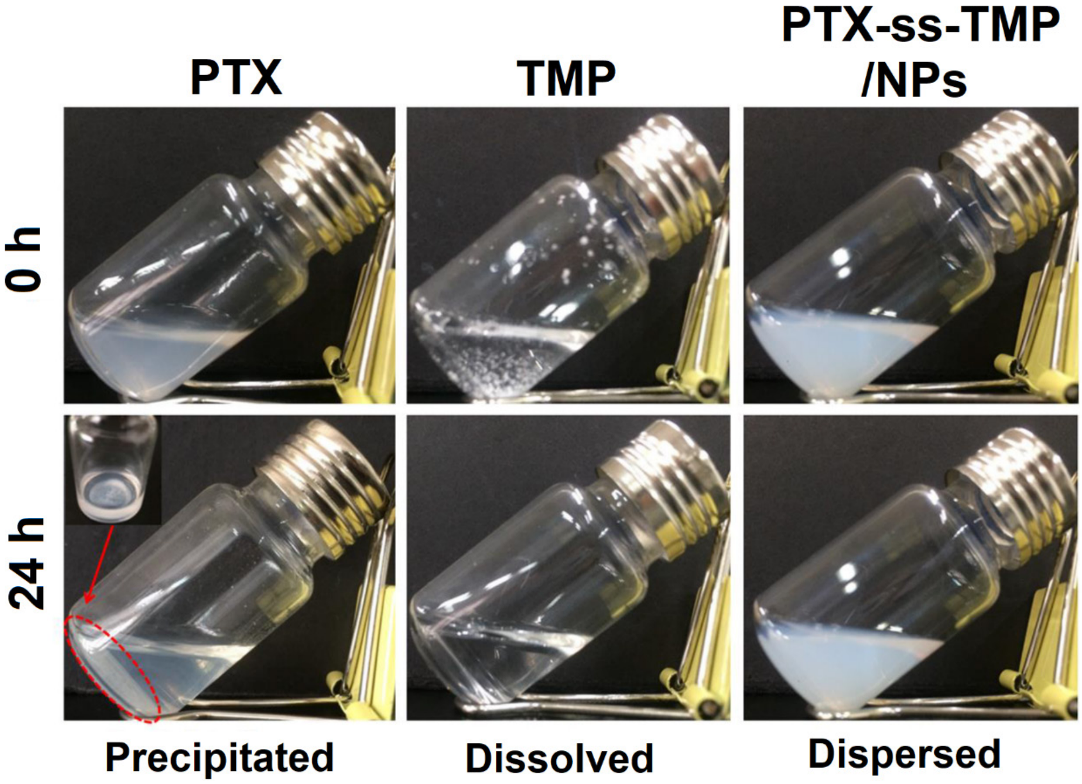

Figure S6. Photographs of free PTX (1 mg/mL) precipitated in PBS, free TMP (1 mg/mL) slightly dissolved in PBS, and PTX-ss-TMP NPs (2 mg/mL) dispersed in PBS.

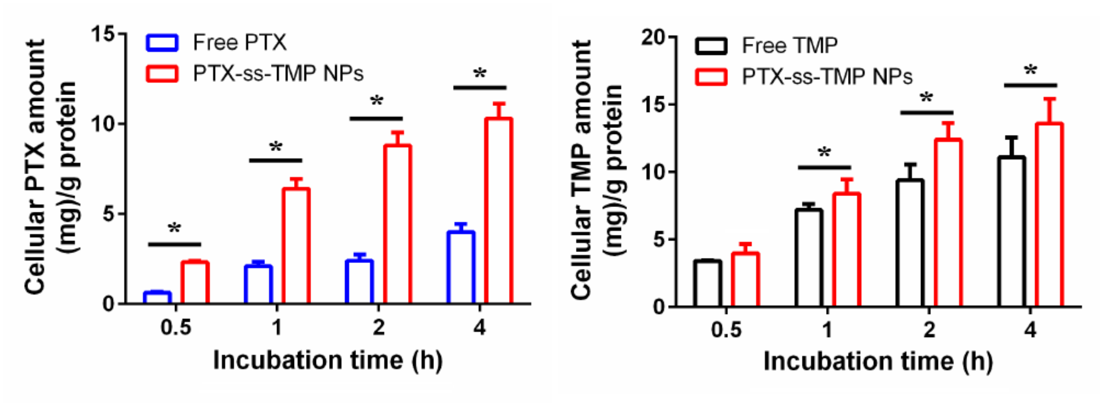

Figure S7. Intracellular uptake of PTX and TMP in A2780 cells after treatment with free PTX/TMP or PTX-ss-TMP NPs for 0.5, 1, 2, 4 h, respectively. PTX or TMP absorbed in cells was determined by HPLC method followed by a series of approaches descripted in Method Section.

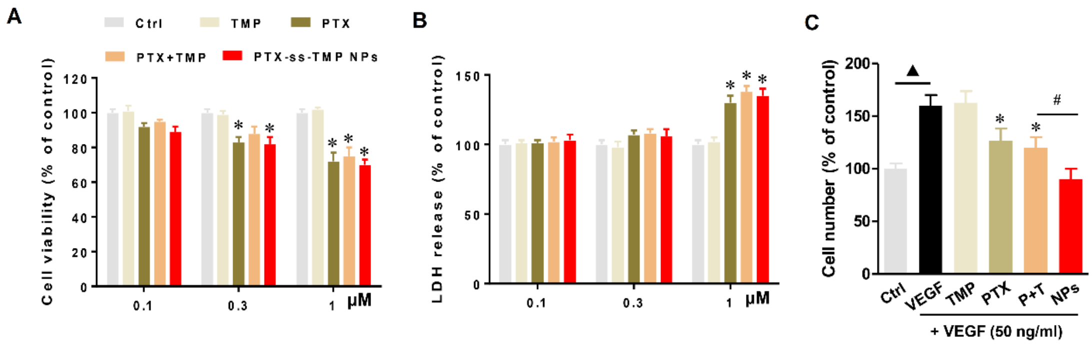

Figure S8. Effects of PTX-ss-TMP NPs on angiogenesis in vitro. (A) Effects of various formulas (free TMP, free PTX, PTX+TMP mixture, and PTX-ss-TMP NPs) at 0.1, 0.3, and 1 μM on HUVEC viability for 48h treatment. Cell viability was evaluated by MTT assays. (B) LDH release in HUVECs caused by various samples as above-mentioned. (C) Cell growth inhibition of various formulas at 0.1 μM based on VEGF stimulation.

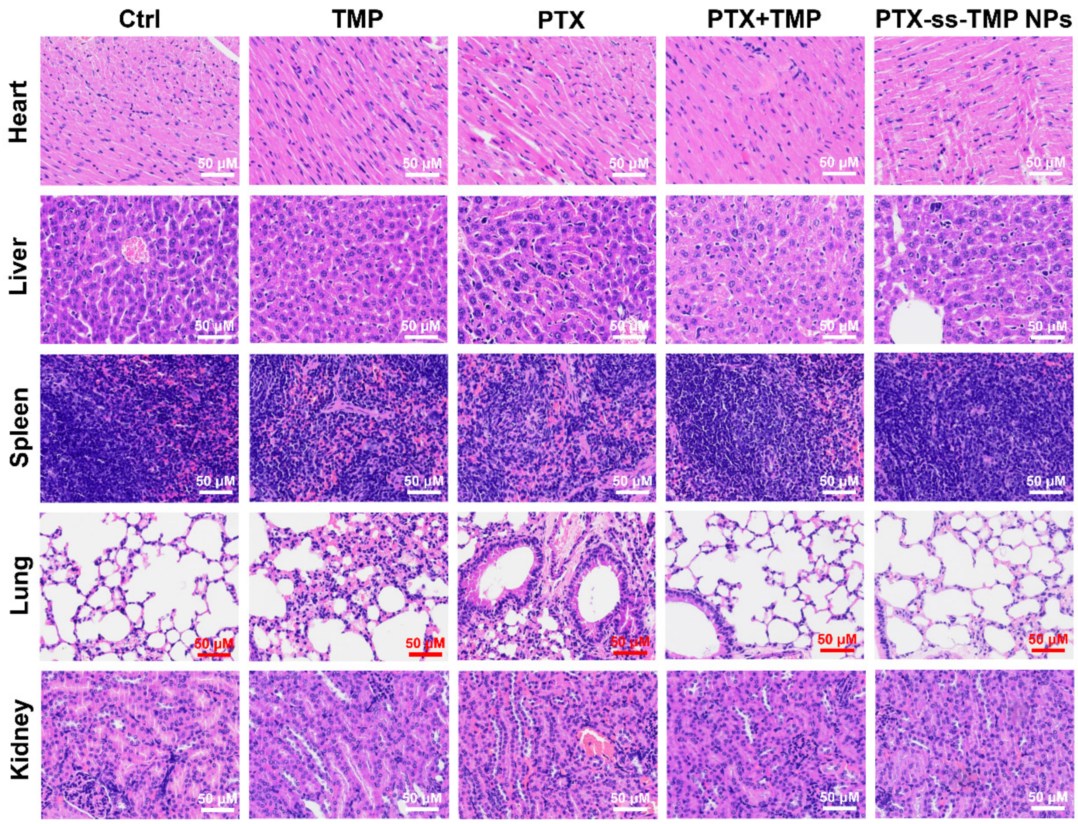

Figure S9. Histopathological analysis by hematoxylin and eosin (H&E) staining of heart, liver, spleen, lung, and kidney sections isolated from nude mice after treatment with saline, TMP, PTX, PTX and TMP mixture, and PTX-ss-TMP NPs, respectively.
